# Study on the Pakistan stock market using a new stock crisis prediction method

**DOI:** 10.1371/journal.pone.0275022

**Published:** 2022-10-20

**Authors:** Irfan Javid, Rozaida Ghazali, Irteza Syed, Muhammad Zulqarnain, Noor Aida Husaini

**Affiliations:** 1 Faculty of Computer Science and Information Technology, Universiti Tun Hussein Onn Malaysia, Parit Raja, Malaysia; 2 Department of Computer Science and Information Technology, University of Poonch, Rawalakot, AJK, Pakistan; 3 Faculty of Computing, The Islamia University of Bahawalpur, Bahawalpur, Pakistan; 4 Faculty of Computer and Information Technology, Tunku Abdul Rahman University College, Kuala Lumpur, Malaysia; Sant Longowal Institute of Engineering and Technology, INDIA

## Abstract

A Stock market collapse occurs when stock prices drop by more than 10% across all main indexes. Predicting a stock market crisis is difficult because of the increased volatility in the stock market. Stock price drops can be triggered by a variety of factors, including corporate results, geopolitical tensions, financial crises, and pandemic events. For scholars and investors, predicting a crisis is a difficult endeavor. We developed a model for the prediction of stock crisis using Hybridized Feature Selection (HFS) approach. Firstly, we went for the suggestion of the HFS method for the removal of stock’s unnecessary financial attributes. The Naïve Bayes approach, on the other hand, is used for the classification of strong fundamental stocks. In the third step, Stochastic Relative Strength Index (StochRSI) is employed to identify a stock price bubble. In the fourth step, we identified the stock market crisis point in stock prices through moving average statistics. The fifth is the prediction of stock crises by using deep learning algorithms such as Gated Recurrent Unit (GRU) and Long-Short Term Memory (LSTM). Root Mean Square Error (RMSE), Mean Squared Error (MSE) and Mean Absolute Error (MAE) are implemented for assessing the performance of the models. The HFS-based GRU technique outperformed the HFS-based LSTM method to anticipate the stock crisis. To complete the task, the experiments used Pakistan datasets. The researchers can look at additional technical factors to forecast when a crisis would occur in the future. With a new optimizer, the GRU approach may be improved and fine-tuned even more.

## Introduction

Predicting stock crisis fluctuations is challenging due to the unpredictable nature of stock values. The stock price crisis is defined as a major decline in the stock price of more than 10% in a few days as a result of excessive selling [1]. There are several causes behind the stock market’s recent drop: a) The Company’s stock is expensive, b) The Company reports a loss, (c) The trade war has caused a global market slump, (d) Tensions in the geopolitical sphere, (e) Pandemics, such as Corona Virus Infectious Disease 2019 (COVID-19).

Trading becomes more beneficial when stock values can be predicted ahead of time [2]. Traded asset values, such as stock prices, already represent all publicly accessible data, according to the Efficient Market Hypothesis [3]. A significant number of research studies [4–7] give data that contradicts the Efficient Market Hypothesis’s claims. These findings suggest that the prediction of the stock market is possible to some extent. Forecasting of the Stock crisis assists financiers in exiting the market at an appropriate spell. A crisis in the *n* stock market might be triggered by changes in the economic policy and macroeconomic statistics of the globe or any particular place. As, in 2008, a financial market slump [8] began in the United States (US) and has subsequently spread to other countries’ economies. It’s been noted that the crisis might begin in a developed economy, with the crisis’ influence spreading to growing economies. Like, the crisis of subprime mortgages initiated in the United States and grew into the crisis of sovereign debt in Europe. The Asian market was also hit by the crisis. Prediction of crisis has been widely used in the banking sector, investing, business, and other industries. The importance of crisis prediction for the financial sector drew the attention of many academics and researchers. The capacity to forecast a catastrophe is one of the most significant contributions of the proposed work.

Using deep learning classification algorithms, Chatzis et al. [9] suggested stock crisis occurrences. The research defined a stock crisis as less than one percentile drop in stock returns. Because strong fundamental equities perform better under technical analysis, this study was not deemed a fundamental examination to determine stock quality. Before a stock falls, it generates a bubble. Without taking into account the stock market bubble, the analysis predicts a stock market crash. As a result, one of the goals of this research is to identify stock bubbles.

The future of stock trading is being shaped by Artificial Intelligence and machine learning algorithms. Robo-adviser uses artificial intelligence to evaluate massive amounts of data, execute transactions at the best price, anticipate markets more accurately, and effectively manage risk so that investors receive higher returns. Recently, machine learning approaches have been used in research to identify and forecast time series data. A machine learning model uses data from previously unexplored datasets to detect patterns or make decisions. For instance, to compare the extent of the vegetable and the NDVI’s computing cost, a machine learning-based Random Forest approach was employed [10]. In order to estimate the ice thickness on glaciers, ANN coupled with remote-sensing techniques is used [11]. The study [12] uses SMOTEDNN to address air pollution classification. To determine the probability that permafrost would be distributed along transects under observation, logistic regression models were used [13]. According to the current research, neural network models have played a significant role in predicting and dealing with numerous classification problems. Deep learning techniques have significant importance in the time series domain for the extraction of useful knowledge. Deep learning algorithms have been developed to overcome the limitations of standard neural networks. It’s a sophisticated method that’s been used in a variety of applications, including groundwater storage change [14], understanding of climate variables [15], weed detection [16], climate change forecasting [17], transfer learning [18], and computer vision [19]. The use of deep learning techniques is motivated by the low cost of computer equipment, robust processing capabilities, and a high degree of innovation in machine learning techniques.

Stock price crises are difficult to spot since they can result in a market financial crash, a COVID-19 medical emergency, and geopolitical turmoil. Therefore, predicting future stock values is difficult. As a result, it provides an opportunity to research stock market concerns.

This study makes the following contribution:

1. The Hybridized Feature Selection (HFS) method was proposed to eliminate extraneous financial ratio characteristics. The HFS approach is used for the first time to anticipate stock market crises.
2. To select a strong fundamental stock, the Naïve Bayes classification approach is used.
3. The StochRSI technique is used to identify stock price bubbles. StochRSI is commonly used to identify overbought and oversold movements. The overbought technique is used to identify stock price bubbles for the first time.
4. Moving average statistics are used to identify stock market crises.
5. The LSTM and GRU algorithms are used to forecast future stock price crises. The GRU is being used for the first time to predict stock market crises in this study.

The following is the outline of the paper: The second section discusses relevant research on stock market forecasting. The HFS-based stock crisis forecasting model has been presented in Section 3. In section 4 of the paper, the experimental outcomes and discussion are presented. In section 5, we wrap up by discussing the suggested technique in detail.

## Literature review

The study [20, 21] addressed the importance of determining the link between stock price and exchange rate. The factors were shown to be negatively linked in the study. As a result, the least square estimator is incapable of determining an appropriate link between the stock market and the exchange rate. For the solution to this particular problem, a quantile regression technique has been proposed by the authors. Another study [22] provided a refined structure for the daily based stock price and trading predictions which relies on return distribution and volatility. According to this research, the Auto-Regressive Conditional Heteroscedasticity (ARCH) model is difficult for forecasting when relies on asset return distribution and volatility because of negative correlations (Andersen et al., 2003).

The Support Vector Machine (SVM) model was introduced by the author [23] to anticipate stock values. The experiment used daily stock price data from the Korean composite stock price index. The study took into account two variables based on stock price movements, such as 1 implies up and 0 represents down. A total of 2928 trade samples were evaluated, with 20% of the data being utilized for holdout and 80% for training. Normalization [-1.0, 1.0] was used to scale the original data. The SVM model is provided input data from 12 technical indicators to complete the task. The upper bound C of SVM parameters and the kernel parameter sigma square are investigated in this study.

A prominent strategy for stock price categorization and pattern discovery is to use an Artificial Neural Network (ANN). Most modern applications use ANN to create intelligent and clever computers for science and business. The ANN learns from previous patterns and utilizes that knowledge to predict future patterns. As nonlinear data can be handled through ANN without understanding the link between the input data and the output data, the ANN model is versatile. Deep neural networks are further investigated in study [24, 25].

In a time-series application, the Autoregressive Integrated Moving Average (ARIMA) model has been used widely for finding the linear connection. ARIMA model, on the other hand, is unable to detect nonlinear patterns in data, according to the majority of academics. As a result, SVM and ANN were used in the majority of the methodologies. The study [26] suggested hybridized ARIMA and SVM models for stock price prediction. ARIMA is used to get the residuals, which are then fed into SVM for prediction. Two factors were included in the study [27]: daily stock returns and the volatility index (VI). To determine the degree of correlation between daily returns and VI, the ARCH model is used. This study finds that VI outperforms daily returns for anticipating volatility.

A hybridized artificial intelligence system for forecasting the stock price was proposed in the study [28]. To forecast daily stock prices, this method gathers a neural network and a rule-based system. Backpropagation and perceptron are used to compare the outcomes. The stock of the S&P 500 is used in the experiment. In comparison to Backpropagation, reasoning neural networks have a faster learning rate and fewer hidden nodes.

A Wavelet Denoising-based Back-propagation network (WDBP) was proposed for predicting stock prices. The performance of the WDBP model has been calculated using the MAE, and RMSE measures. From 1993 through 2009, data from the Shanghai Stock Exchange was used in the experiment. The information is divided into binary categories: the first one is training and the second is testing. Training utilized 80% of the data and 20% has been used for testing. The wavelet transformation is used to break data into several layers in this method. Low-frequency or high-frequency signals can be produced. The wavelet transformation frequency is used to estimate the future value of a Backpropagation neural network [29].

The Support Vector Regression (SVR) was used in the majority of studies to forecast stock prices [30–32]. One of the most difficult aspects of SVR is estimating the kernel function’s parameters. Parameter estimate is manually done currently, i.e. through trial and error, in existing work. But, this hand calculation is incorrect. The authors presented different kernel learning approaches to improve the SVR parameters to avoid this issue [33].

A structure for portfolio management was investigated through a model of linear, cubic, and quadratic curves in the study [34]. The research divides various stocks of industries into clusters and the R-squared metric evaluates the model’s performance. Stock prices were predicted using online textual news [35]. The news impressions were classified using Nave Bay’s classifier.

The study [36] employed Delay Neural Networks (TDNN), Back Propagation Neural Networks (BPNN), and Radial Basis Function Neural Networks (RBFNN) to estimate market values. The BPNN model outperformed the other models, according to the research. Another study [37] used the ANN approach to examine stock price prediction. The RMSE measures were used to evaluate the model’s performance.

The Elman Neural Network (ENN) model was used to anticipate stock prices in the research [38], and the ENN model’s parameters were optimized using the GreyWolf optimizer approach. Furthermore, the DNN model outperforms the SVM and ANN approaches [39]. DNN includes three layers of neural networks more than that of ANN, which allows the model to learn more precisely.

Table 1 summarizes the entire relevant work. The majority of the research focused on stock price forecasting [40–44]. Only a little amount of research has been done on stock market crisis prediction. The study [9] presented a categorization approach for stock crisis prediction. For stock crisis detection, the Log-Periodic Power Law (LPPL) approach was used [45, 46]. As a result, it provides a chance to investigate stock crisis-based forecasting.

**Table 1:** Related work.

Author	Techniques	Dataset	Evaluation Metrics	Advantages & Disadvantages	Outcome
Chong Li [45]	LPPL	Shanghai Shenzhen CSI 300 index.	Lomb-Scargle periodogram	The LPPLS model successfully recognized the bubble characteristics of faster-than-exponential rises and falls in the Chinese stock market index, according to the back-tests of bubbles.	Chinese Market Bubble identification
Chenn Huanget et al. [47]	SVM, ANN	Korea and Taiwan stock Exchange.	Wrapper Method	The experiment’s findings indicate that the voting plus wrapper technique can accurately forecast events up to 80.28% of the time.	Stock Price Classification
Mehmet Orhan et al. [48]	GARCH	MSCI (Turkey, Brazil, USA, and Germany)	Kupiec test	NPGARCH and NPGARCHK exhibit worse performance. The research will be used to determine which GARCH specification will produce more accurate VaR estimations and be more statistically significant.	Stock Index Prediction
Sotirios P Chatzis et al. [9]	DNN	FRED database & SNL website.	G-mean Discriminant power Balanced accuracy	The results show that, in comparison to other methodologies, deep learning techniques can achieve an unprecedented level of accuracy.	Stock Crisis Classification
Jan Wosnitza et al. [46]	LPPL	CDS spreads	Mann-Whitney U test	The qualitative data analysis shows that throughout the global financial crisis, CDS spread fluctuations followed LPPL trends.	Liquidity Crisis
David Enke et al. [49]	Neural Network	S&P 500 index	RMSE	The findings demonstrate that on S&P 500 index trading, classification models may provide greater returns than level estimation techniques.	Stock Price Prediction
Chandar [36]	TDNN, RBFNN, and BPNN	Yahoo! finance	RMSE, MAPE MAE, MSE	The experimental findings show that, for nearly all of the stocks, BPNN consistently outperforms other models, including TDNN and RBFNN, in terms of all assessment criteria.	Stock Price Prediction
Nikolay Y. Nikolaev et al. [50]	GARCH	DEM/GBP series	NMSE, NMAE	Regarding log-likelihood and a number of statistical features, the nonlinear NGARCH (1, 1) performs better than both the GARCH (1, 1) and TGARCH (1, 1) linear models.	Currency Volatility Prediction
Chandar [38]	Elman Neural Network	NYSE and NASDAQ stock data	MSE, RMSE, MAE, SMAPE	It appears that by displaying lower values for all metrics in nearly all the equities, the GWO-ENN offers promising outcomes.	Stock Price Prediction
Maji et al. [34]	Linear, Quadratic, and Cubic curve model	Bombay Stock Exchange	RMSE, R-squared value	The advantage of the suggested methodology is that it is less reliant on professional fund managers timing their buys and sells in the market.	Portfolio Management Framework

## Methodology

The LPPL approach was suggested by the study [11] to identify stock price bubbles. The term "bubble" refers to the exponential rise in stock values. This research was not conducted as a fundamental examination to determine the stock’s quality. After the 1929 stock market crisis, the author [30] lost money. He then published a book on basic stock analysis [31]. According to the study [30], the fair value of the price for the stock is determined by the profits, asset worth, and dividends of the company. As a result, we’ve looked at financial indicators to determine stock quality.

**Fig 1:**
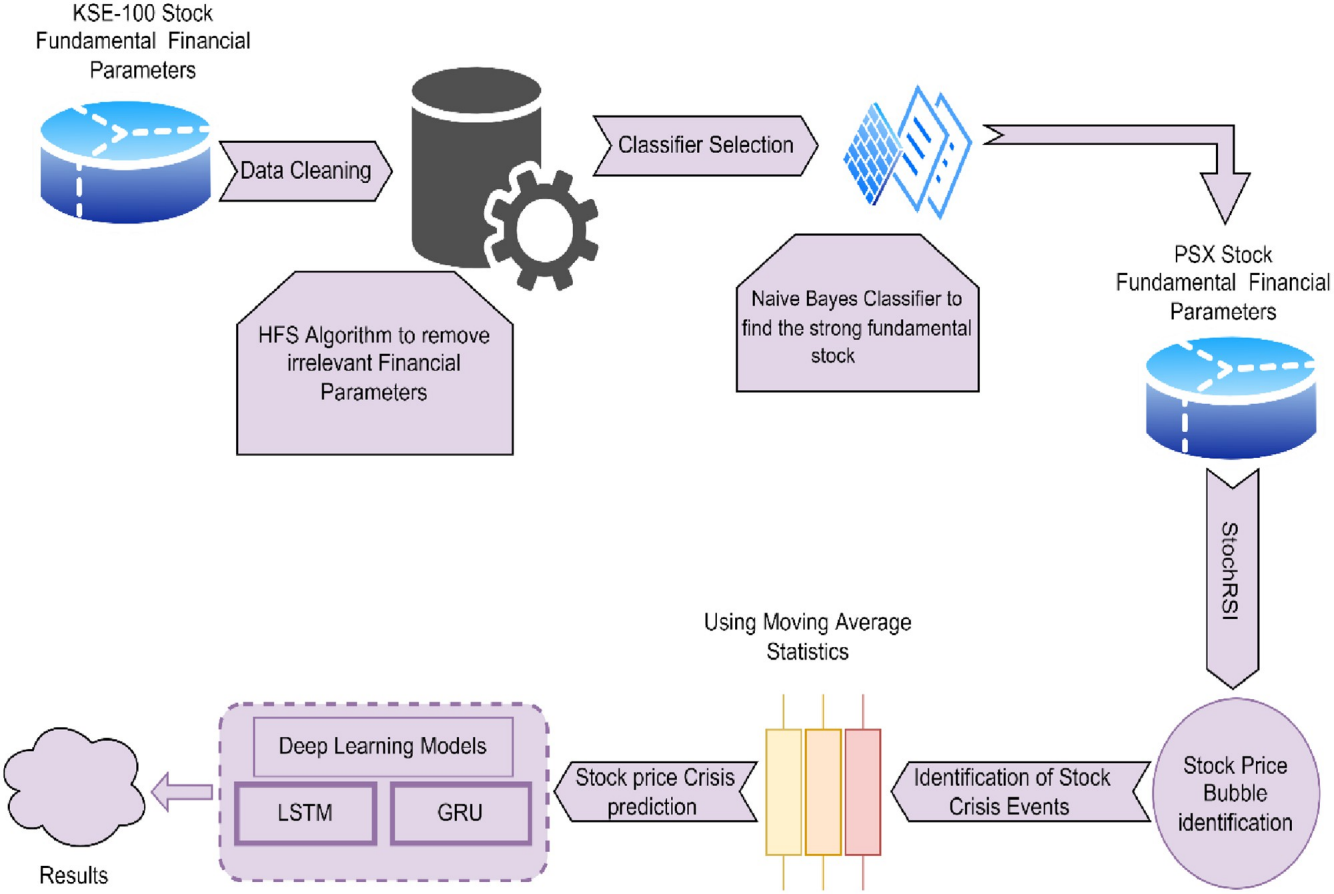
Proposed model for stock price crisis prediction.

Fig 1 depicts the general flow of the planned task. Using the LSTM and GRU algorithm, we devised a Hybridized Feature Selection approach for anticipating forthcoming stock value crises.

### A. Removing irrelevant features by using hybridized feature selection approach

**Fig 2:**
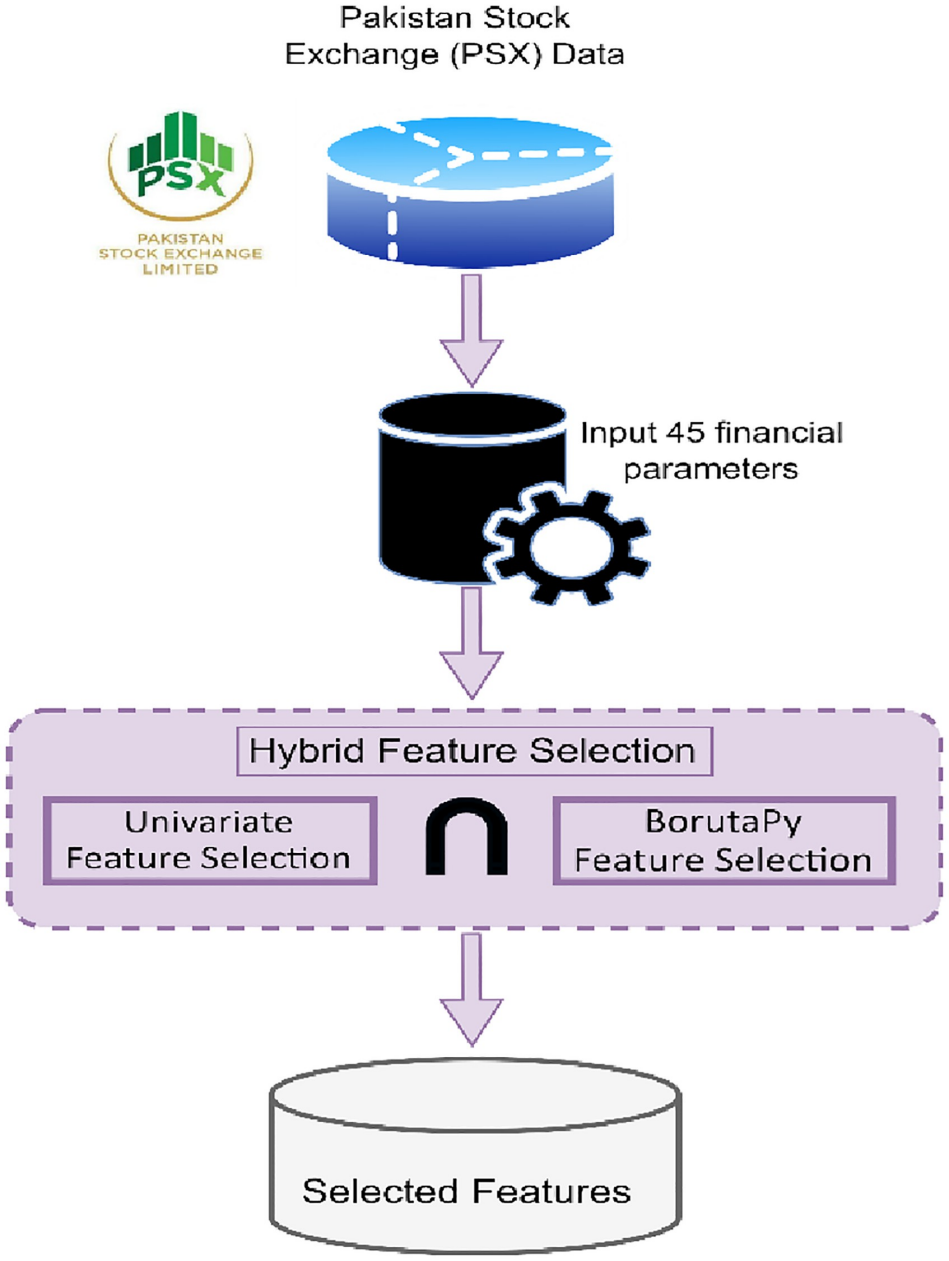
Hybridized feature selection model.

The fair value of a stock price is determined by the stock’s financial criteria. There are other financial characteristics to consider, such as price to earnings, company returns, corporate debt, and so on. Identifying meaningful stock metrics is a difficult process. As a result, we introduced the Hybridized Feature Selection (HFS) approach for selecting important financial parameter attributes. The HFS approach integrates two distinct algorithms: Univariate Feature Selection and BorutaPy Feature Selection. The outcome of this combination was likewise subjected to an intersection operation. Fig 2 depicts the suggested work. Algorithm 1 describes the suggested HFS approach in detail.

**Algorithm #1.** Hybridized Feature Selection Algorithm.

1: Set 45 financial stock parameters.

2: Use feature selection algorithms such as UFS and BorutaPy to find the best features.

3. The following is the UFS algorithm:

4. Select k Best (k highest scoring features) for each financial characteristic feature.

5. Eliminates all except a user-specified percentage of the highest-scoring attributes.

6. Select False positive rate.

7. Select False discovery rate.

8. OR Select Family wise error

9. Select Generic Univariate

10. Retain the feature k of the essential financial parameter and eliminate the weak financial characteristics.

11. end UFS

12. As follows is the BorutaPy algorithm:

13. The dataset is duplicated, with the values in each column shuffled. Shadow characteristics are the names given to these variables.

14. Duplicate or shadow the financial parameter.

15. To discover key financial parameter characteristics, train the random forest classifier.

16. Compare the original feature’s Z score against the shadow feature’s Z score in each iteration.

17. The features with the lowest Z score must be removed.

18. end BorutaPy

19. Execute UFS ∩ BorutaPy operation.

20. Output Best Features.

**Fig 3:**
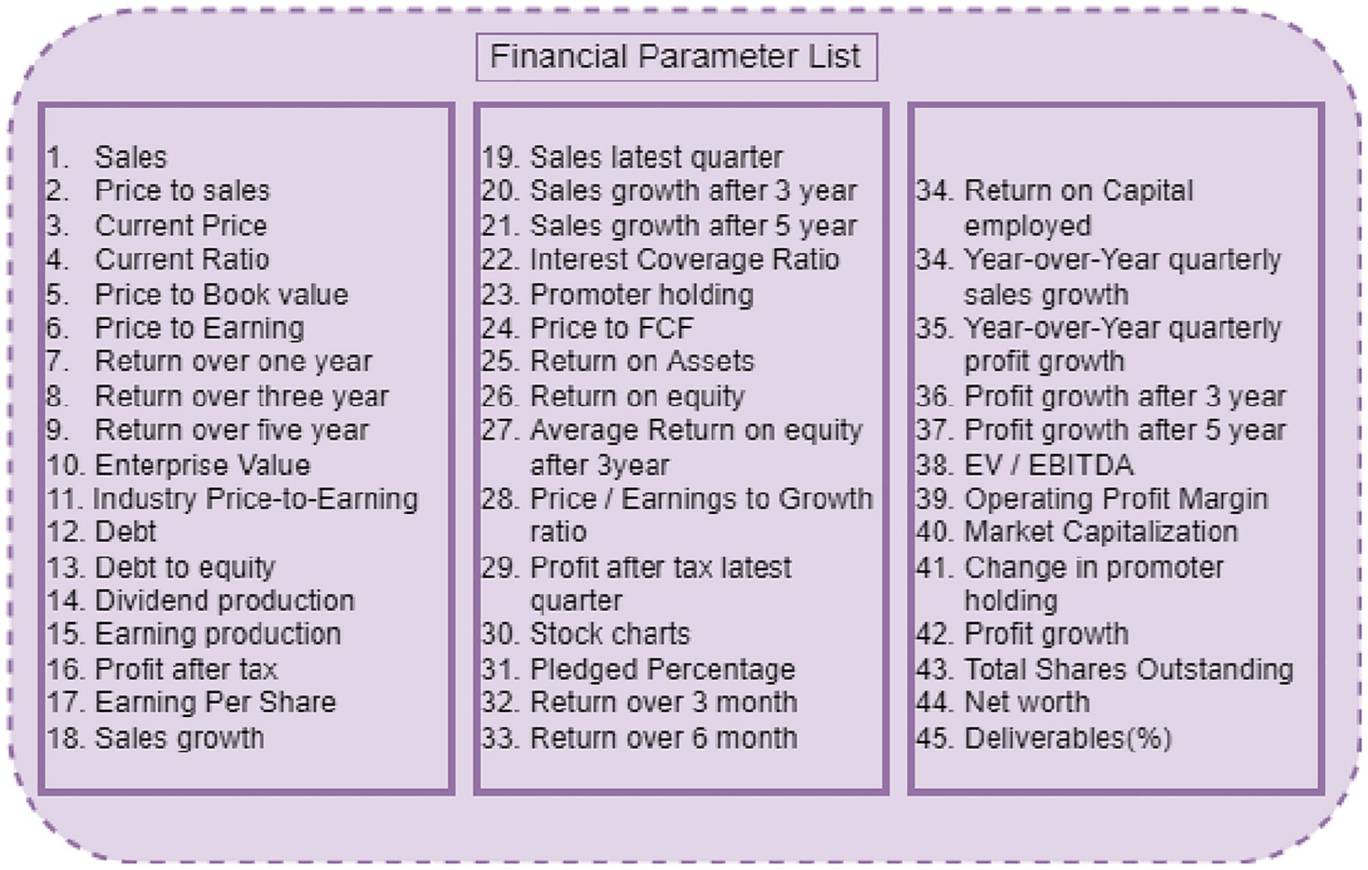
Financial parameters of the stock market.

**Fig 4:**
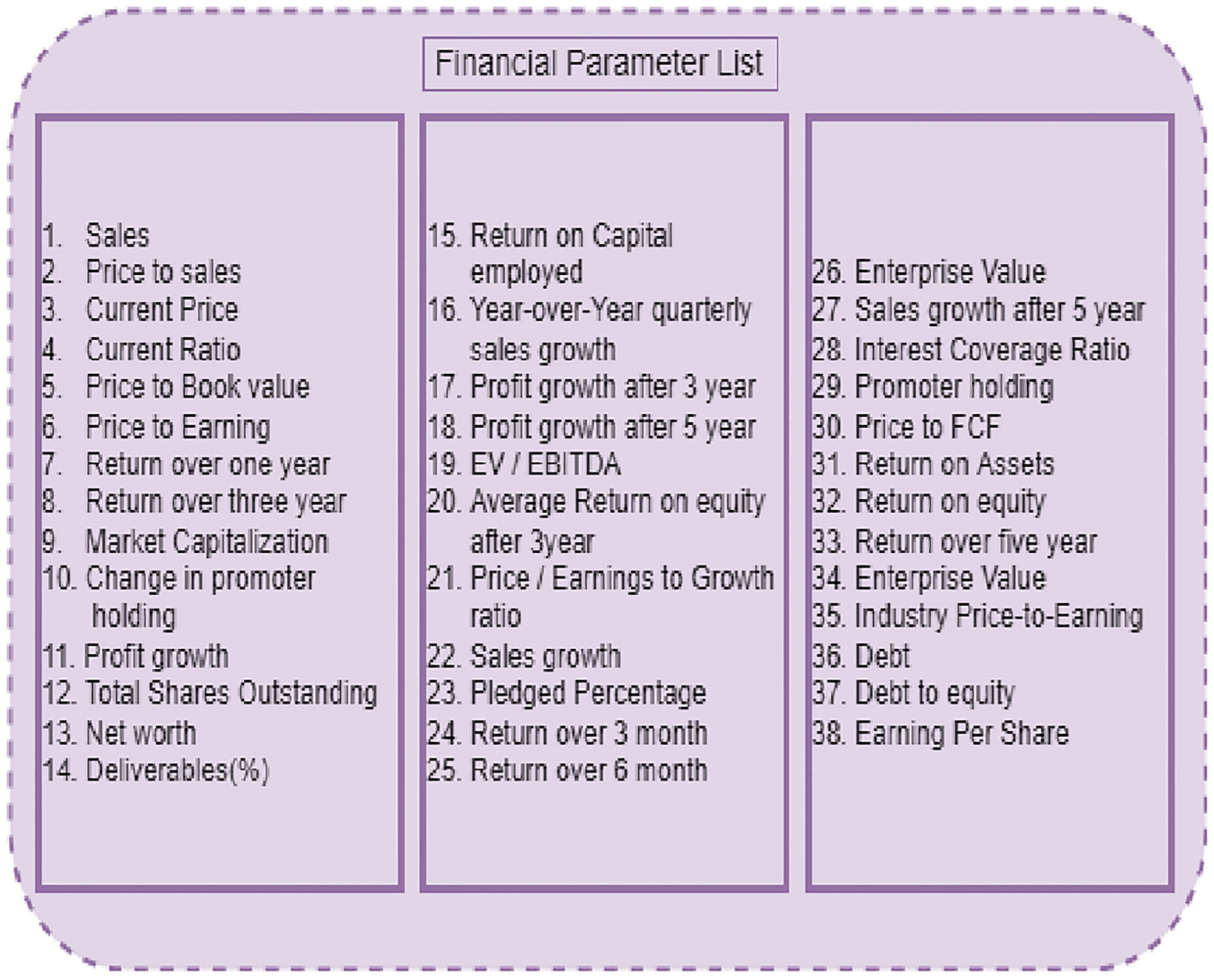
UFS based final selected features.

We used Karachi Stock Exchange-100 index (KSE-100) stocks to carry out the set of trials. The financial statistics of the KSE-100 stock are acquired from the Pakistan Stock Exchange (PSX) [51]. Fig 3 depicts the list of stock financial attributes. We examined 45 different KSE-100 stock financial attributes/parameters. The next step is to use the Univariate feature selection (UFS) approach to discover important stock financial parameters. To pick the best feature, the 45 stock financial features are fed into the UFS algorithm. UFS selects the best features from a set of bivariate or univariate statistical tests. In sklearn, there are several univariate feature selection approaches; we’ll focus on the SelectKBest method, which is the most often used. The SelectKBest technique is used, but the score function is also required for each function. For regression, f_regression, and mutual_info_regression are commonly used and chi^2^, f_classif, and mutual_info_classif are often used for classification. These functions employ several tests: f_regression employs univariate linear regression tests, f_classif employs the Analysis of Variance (ANOVA) F-value approach and chi^2^ k-implements chi-square statistics. Mutual_info_regression and mutual_info_classif are functions that use k-nearest neighbors’ distances to estimate entropy. Apart from the score function, SelectKBest has another argument, k. SelectKBest calculates scores using the score function and selects k features at a time. The characteristics are labeled as True or False by SelectKBest. We will get the best features using the True tag (strongest relationship to output). The target variable here is the price-to-earning (P/E) financial parameters. According to the UFS technique, 38 attributes are found to be important, as shown in Fig 4.

**Fig 5:**
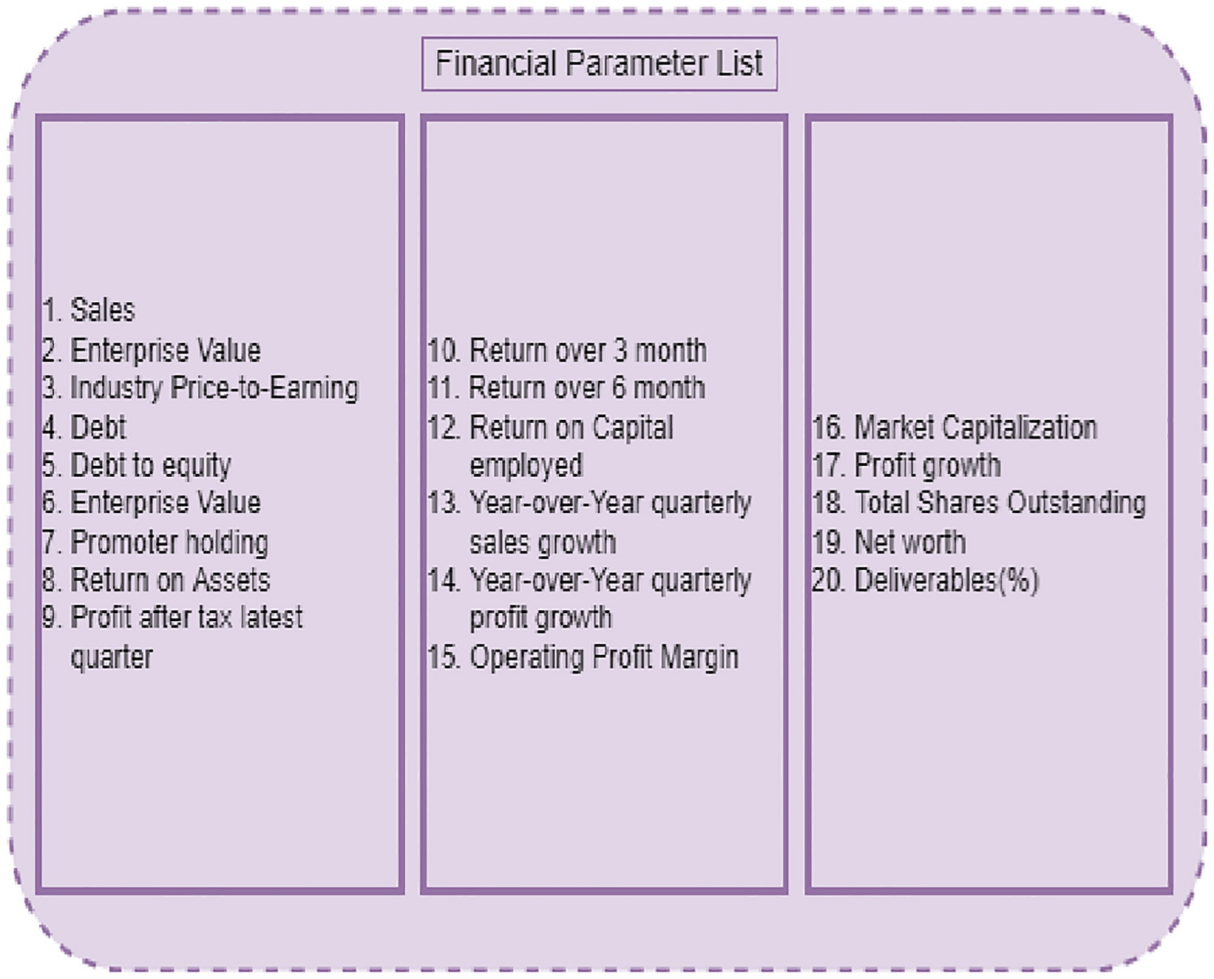
BorutaPy feature selection algorithm based final selected features.

To eliminate the irrelevant feature, the BorutaPy feature selection approach is utilized. The 45 stock financial parameters are fed into BorutaPy, which then chooses the optimal features. BorutaPy works by creating shadow financial parameters, duplicating the dataset, and shuffling the column values. The target variable is the financial parameter Price to Earnings (P/E) which has been used for regression. The next following step is to use random forest regression to train the model and uncover significant financial parameter features. The final 20 features are identified as significant using the BorutaPy approach and are shown in Fig 5.

**Fig 6:**
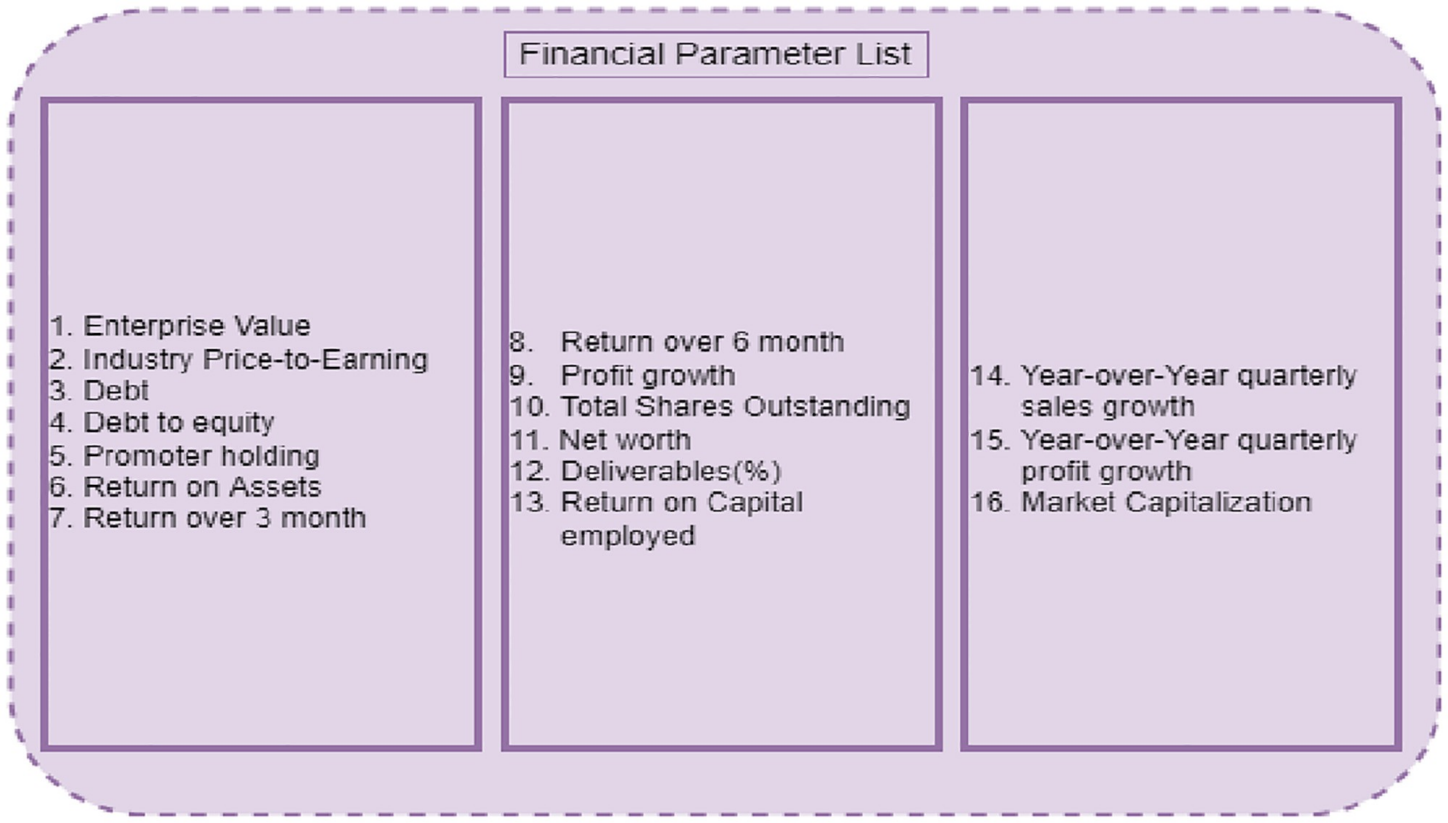
HFS based final selected features.

An intersection operation has been conducted by the authors on the results of UFS and BorutaPy feature selection methods. Lastly, the authors acquired 16 characteristics, which are shown in Fig 6. The quality of stock has been categorized by employing the Naive Bayes which uses the final selected features as input, obtained from the HFS method.

### B. Naïve bayes classification approach

To detect positive and negative attitudes, in text categorization and sentiment analysis, Nave Bayes is extensively employed [52–54]. This study uses the NB classification technique to choose the strongest fundamental stock which relies on financial data. The target variable is the financial parameters of price to earnings (P/E) i.e., the likelihood of p (stock quality). The target variable is then used for finding the frequency against each financial stock parameter individually. Eq 1 defines the likelihood of stock quality (SQ) and Financial Parameters (FP). A stock with a higher possibility of being fundamentally strong is examined here. The highest probability of fundamentally strong stock is analyzed from KSE-100 stock in the tests which rely on the Naive Bayes classifier.


$$P\left(\frac{FP}{SQ}\right)=\frac{P\left(\frac{SQ}{FP}\right)P\left(FP\right)}{P\left(SQ\right)}$$ (1)

### C. Stochastic RSI method for identification of stock price bubble

Stock price bubbles are identified using Stochastic Relative Strength Index (StochRSI) statistics. The range of the StochRSI technical indicator is 0 to 100. StochRSI numbers below 20 suggest the oversold stock, while numbers above 80 represent the overbought stock. When the StochRSI indicator value exceeds 80, it indicates that the stock price is likely to decrease. Because of an overpriced stock. The StochRSI was calculated using the first 20 fundamentally strong equities.

The StochRSI value was calculated using historical data of stock price from the Pakistan Stock Exchange (PSX) portal. From 2010 until December 2021, we studied historical stock data. Then, using the equation below, determine the StochRSI value depending on the stock price.

$$\text{StochRSI}\;\left(\#\text{Days}\right)=\text{RSI}–\text{min}\left[\text{RSI}\right]/\text{max}\left[\text{RSI}\right]–\text{min}\left[\text{RSI}\right]$$ (2)

Where relative strength index (RSI) is equal to the Current RSI reading, min [RSI] is equal to the lowest RSI reading over the last number of days and max [RSI] is equal to the Highest RSI reading over the last number of days. The majority of previous RSI calculations were based on 14 days [55, 56]. However, we used StochRSI for 200 days to discover the stock price bubble in our technique. The rationale for this is that 14 days is employed for intraday trading rather than long-term trading. A stock price bubble is nothing more than an overpriced stock. StochRSI statistics are used to capture the bubbles, as seen in Table 2. The following stage is to identify stock crisis areas based on the stock price bubble.

**Table 2:** StochRSI is used to identify stock price bubbles.

Stock Name	StochRSI Value	Bubble Point Date	Bubble Price
Habib Bank	87	15/May/2017	308
	84	22/Dec/2014	215
	81	04/April/2010	108
United Bank	84	12/Dec/2018	295
	78	04/Nov/2013	190
	81	12/Sep/2011	102
National Bank	74	03/Oct/2019	410
	71	10/Aug/2017	327
	79	06/Jan/2014	205
	80	22/Oct/2011	270
Bank Alfalah	80	21/Aug/2018	169
	83	14/Mar/2014	110
	77	20/Jan/2011	73
Allied Bank	85	15/Jul/2019	205
	79	17/May/2014	133
	77	21/Dec/2011	187
NetSol Tech	85	06/Oct/2020	766
	81	17/Jul/2015	465
Muslim Commercial Bank	77	14/Nov/2020	330
	76	22/Dec/2013	192
Attock Cement Pakistan Limited	76	17/Jun/2015	210
	83	12/Feb/2012	162
Tri-Star Power Limited	71	05/Nov/2019	205
	79	26/May/2016	234
Tri-Star Polyester	81	19/Mar/2015	308
	84	27/Nov/2013	282
The Resource Group Pakistan Limited	74	03/Aug/2020	195
	72	04/Oct/2017	152
Towellers Limited	81	27/Sep/2019	271
	83	14/Feb/2016	166
Service Industries Limited	78	04/Mar/2020	211
Saritow Spinning Mills	71	26/Sep/2017	266
	74	16/Mar/2015	234
Shakarganj Limited	81	27/Nov/2020	235
	85	14/Dec/2017	192
	83	22/Jun/2015	205

### D. Moving average statistics for highlighting the stock crisis incidents

**Fig 7:**
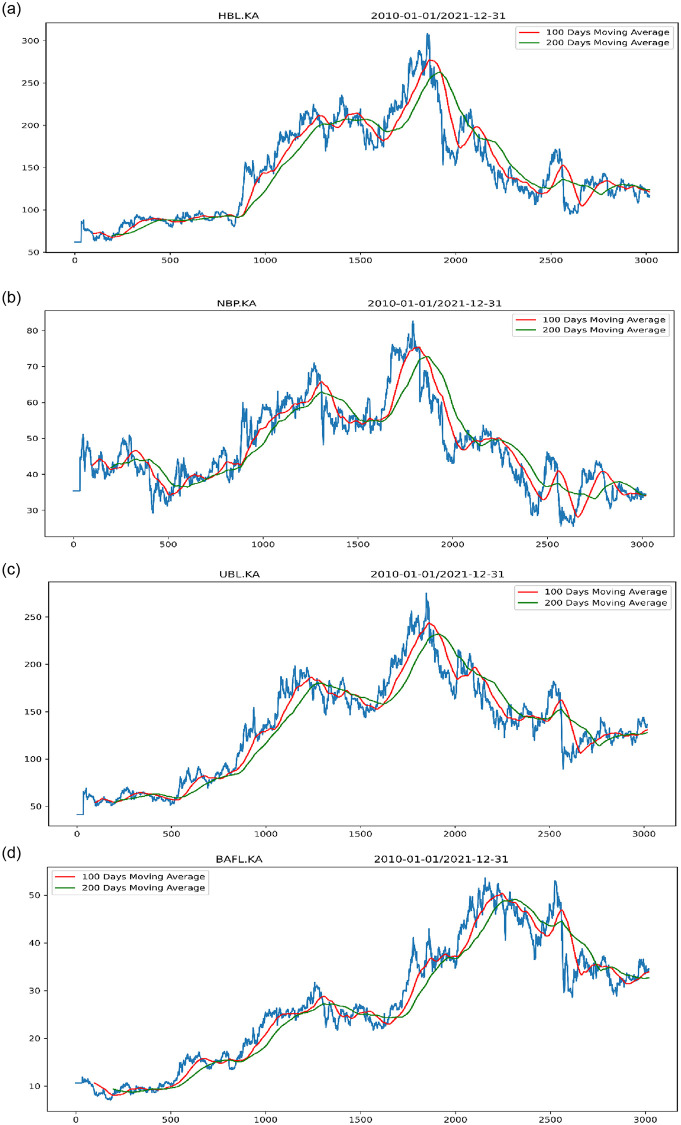
Stock crisis point. (a) HBL Bank. (b) NBP Bank. (c) UBL Bank. (d) Bank Alfalah.

Following stock price bubble identification, the next stage is to identify a stock price crisis. The moving average approach is used to determine when a stock is in a state of crisis. We glanced at the 100-day and 200-day moving averages. The stock price is used to calculate the moving average. The first evolving average of 100 periodic days presents the price’s short fluctuations of the stock, while the next evolving average of 200 periodic days shows the stock price’s long changes. Stock price short changes are lower than long price movements, indicating a stock price decline. These kinds of data points are referred to as starting points for the stock crisis, as seen in Fig 7. The red line in Fig 7 represents a 100-day moving average, while the green line represents a 200-day moving average. When the 100-day moving average falls below the 200-day moving average, it is considered the beginning of a stock market crisis. The stock market’s crisis moment is depicted in Table 3 using moving average figures. We’ve found the stock market’s low point. The next phase is to use LSTM and GRU deep learning algorithms to forecast future stock crisis points.

**Table 3:** Using moving average data, the stock price crisis point is identified.

Stock Name	Crash Point Date	Crash Price
Habib Bank	11/Jul/2018	310
	18/Nov/2015	209
	08/Mar/2011	174
United Bank	22/Oct/2019	268
	24/Aug/2014	198
	16/May/2012	152
National Bank	11/Dec/2020	365
	24/Jun/2018	302
	11/Sep/2016	262
	18/Mar/2013	232
Bank Alfalah	07/Jul/2020	175
	11/Nov/2017	132
	18/Apr/2013	92
Allied Bank	25/Oct/2020	215
	13/Feb/2016	139
	18/Dec/2013	172
NetSol Tech	09/Mar/2019	646
	11/Nov/2016	395
Muslim Commercial Bank	19/Jan/2019	310
	28/Sep/2016	198
Attock Cement Pakistan Limited	11/Jul/2017	230
	21/Dec/2015	192
Tri-Star Power Limited	21/Mar/2020	195
	04/Sep/2014	166
Tri-Star Polyester	16/Apr/2017	266
	22/Dec/2013	205
The Resource Group Pakistan Limited	18/Marc/2019	189
	27/Jul/2015	132
Towellers Limited	27/Jun/2020	265
	04/Sep/2018	175
Service Industries Limited	11/Apr/2019	211
Saritow Spinning Mills	19/Nov/2016	242
	16/Mar/2015	247
Shakarganj Limited	16/Dec/2019	210
	19/Apr/2016	182
	27/Nov/2014	198

### E. Stock crisis prediction using LSTM model

The LSTM layer in deep learning is made up of recurrently linked memory blocks that are capable of learning long-term dependencies. Such blocks are made up of one or more memory cells that are recurrently coupled as well as Input, output, and forget are three multiplicative units that allow read, write, and reset operations to be performed.

The LSTM design was decided to be 4: 100: 100: 50: 1, which indicates the four neurons which lie in the input layer, three hidden layers, each having 100 neurons, the third layer with 50 neurons, and an output neuron. For the hidden layers, the ReLu activation function was employed, whereas the sigmoid function was chosen for the output layer. The network’s loss function was chosen as the mean squared error (MSE). The network was initially operated for 100 epochs, which resulted in over-fitting. During the training phase, the dropout of neurons in all hidden layers was implemented to avoid the over-fitting problem. The study [57] suggested that dropping out neurons is a good way to prevent the over-fitting problem. The value for dropout was set to 0.3, which indicates that on each iteration, 30% of neurons in hidden layers are disregarded during the forward pass. In the following iteration, the dropped neurons reappear, and another 30% of neurons are removed, and so forth. An early stopping strategy was used to improve the model’s resilience, resulting in the ideal number of epochs necessary for the model to attain the minimal error threshold. The batch size was kept constant at 100.

**Fig 8:**
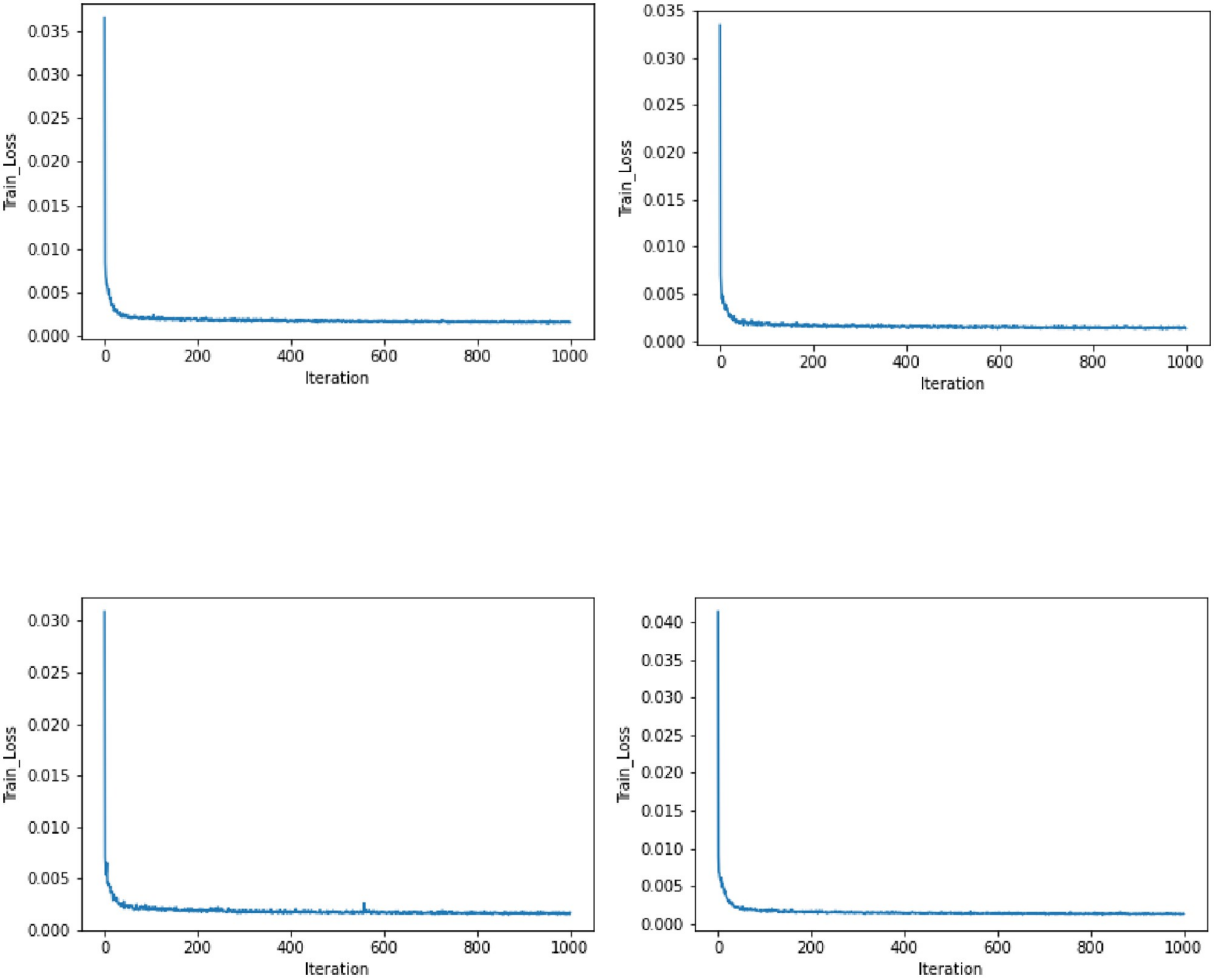
Loss function error rate. (a) HBL Bank. (b) NBP Bank. (c) UBL Bank. (d) Bank Alfalah.

There are 15651 parameters to train for each stock, including connection inputs to each layer as well as bias inputs. Adam’s optimization approach, a stochastic gradient descent model [58], is used by the network. The default learning rate was retained at 0.001. For the first stock, Fig 8 demonstrates an error decrease at each period. The remainder of the equities showed similar behavior. M values must be added back to the LSTM predictions to generate the final predictions. The output of the LSTM for stocks is shown in Fig 11, indicating that the model can accurately capture stock changes.

The program was written in Python and run on an Anaconda environment on a Jupyter Notebook. Important packages like Keras and TensorFlow were employed, resulting in a fast processing speed and good performance.

### F. Stock crisis prediction using GRU model

Cho et al. [59, 60] invented GRU, which is one of the RNN variations. It overcomes the problem of RNN being difficult to deal with long-term information collection by implementing a gating mechanism. GRU is simpler than LSTM, with only an update gate (z_t_) and a reset gate (r_t_) being introduced. The update gate which behaves as an input gate in GRU determines the quantity of input (x_t_) data and prior output (h_t-1_) that should have been transmitted in the next coming cell, while the reset gate determines the quantity of the previous data that should be forgotten. The present memory guarantees the sending of only relevant data in the next iteration that is decided with the help of weight *W*. Eqs 1–6 define GRU network structure. GRU network accepts inputs and adds a bias to the weighted sum of the inputs. A transfer function is used to express this computation. The following equations regulate the major operations of GRU.


$$z=\;\sigma \left(W_{Z}⊙\;x_{t}+\;U_{z}⊙\;h_{t-1}\;+\;B_{z}\right)$$ (3)

$$r=\;\sigma \left(W_{r}⊙\;x_{t}+\;U_{r}⊙\;h_{t-1}\;+\;B_{r}\right)$$ (4)

$$\hat{h}=tanh\left(W_{h}⊙\;x_{t}+\;U_{h}⊙\;h_{t-1}\;+\;B_{z}\right)$$ (5)

$$h=\;\;z*h_{t-1}+\left(1-\text{z}\right)*\;\hat{h}$$ (6)

An activation function uses the estimated sum of weights as an input to create the output. W stands for weight, h and $\hat{h}$ for hidden layer and output, B for bias, and σ for activation function.

The neurons number is set as 8, 16, and 32, the rate of learning is commonly set as 0.001, and the iteration number is set as 1000. By examining the prediction accuracy of experimental results and the degree of fit for the trend between the forecasted price of the stock and the historical price of the stock, we can establish the most accurate prediction technique.

**Fig 9:**
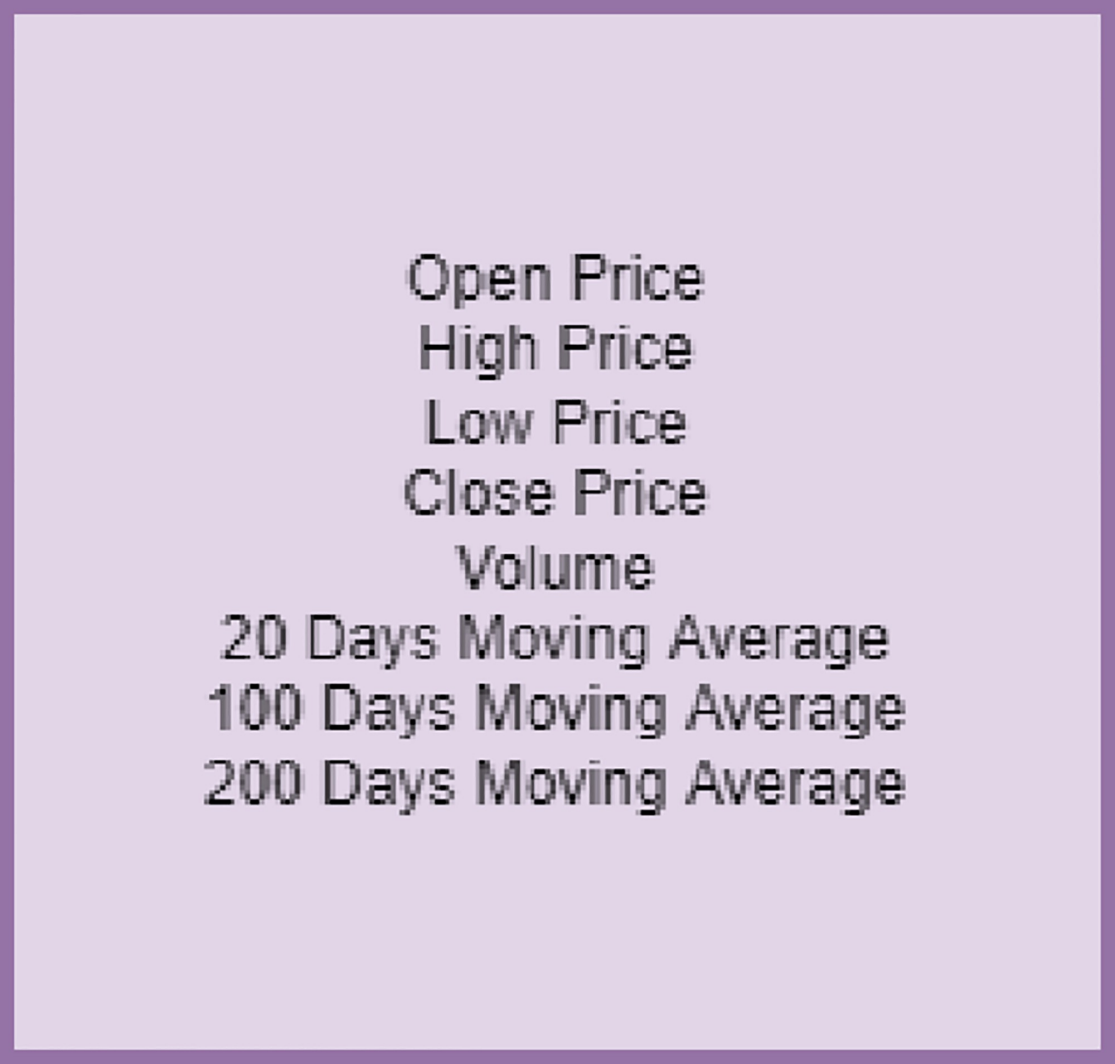
Stock crisis input data.

**Fig 10:**
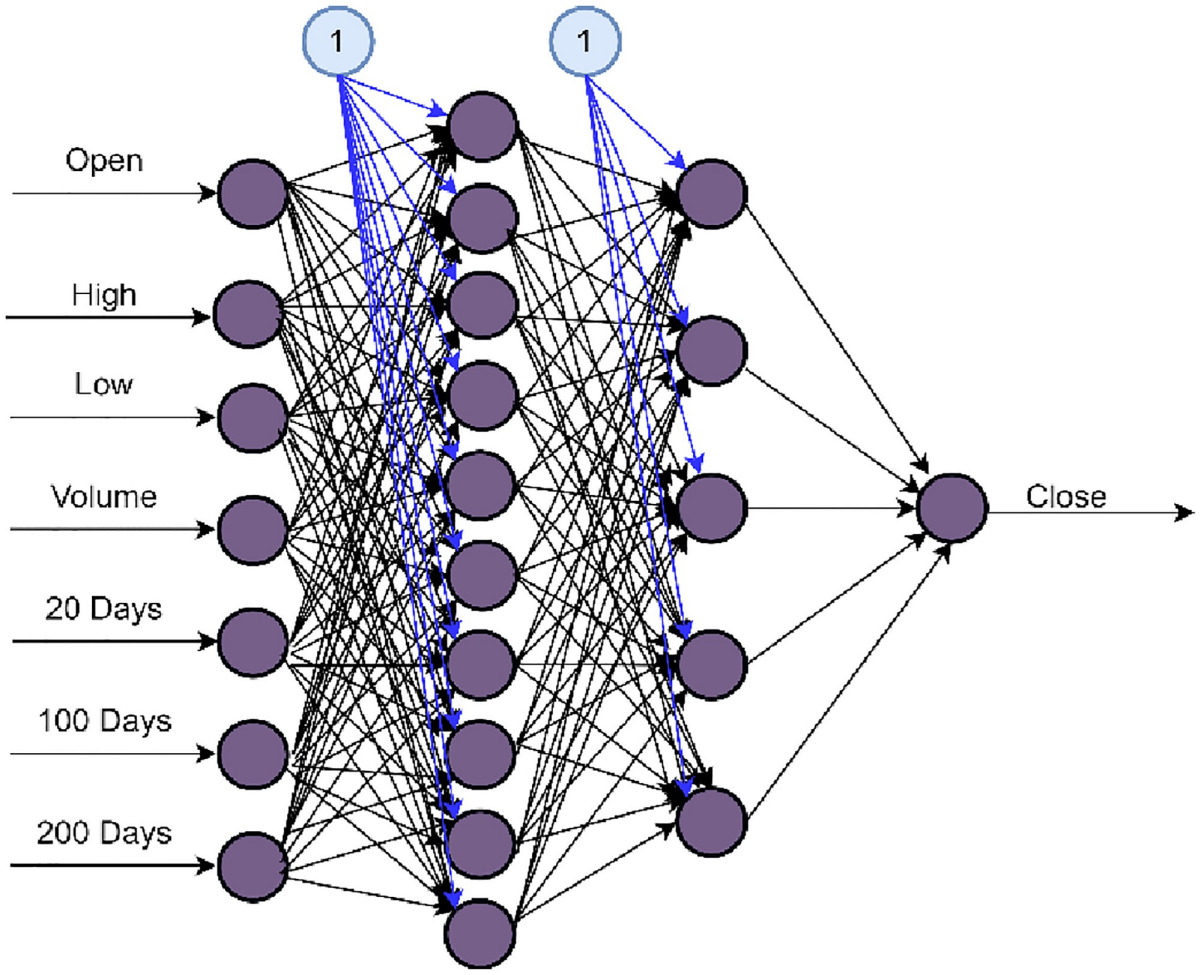
GRU method.

The GRU model is fed data from the stock market crisis. A list of input variables for the stock price is illustrated in Fig 9. Fig 10 depicts the proposed GRU. The target variable is the stock’s closing price which is employed in the GRU model. The mean has been removed from each value and is divided by the standard deviation to normalize independent input variables. In the hidden layer, we use the Rectified linear unit activation function.

## Experiment and result discussion

The application was developed in Python and executed on the Anaconda platform. Because of the stock market’s volatility, identifying a stock crisis is challenging. There are numerous financial parameters to consider, including price to earnings, company returns, company debt, and so on. The challenge of identifying significant stock financial characteristics is difficult. As a result, the Hybridized Feature Selection approach was presented to choose an important financial parameter feature. The stock market crisis was predicted using the LSTM and GRU deep learning algorithms. In this study, we experimented with a few KSE-100 stocks from January 2010 to December 2021. The parameters of the GRU deep learning approach are fine-tuned to achieve the best results. We have varied the learning rate from 0.001 to 0.03. For the LSTM method, the learning rate has increased from 0.001 to 0.03. Ten cross-fold validation has been employed to validate the performance of the model. It is the most often used statistical tool for validating outcomes. This approach divides datasets into two categories: training and test sets, with a test set utilization for assessing the performance of the model. Datasets are separated within ten folds in our tests. The training utilizes 80% of the data, whereas 20% of the data is utilized by testing. We evaluated the results for each cross fold before considering the average of 10 cross folds. The MSE, MAE, and RMSE scores have been used for evaluating the performance of the model which are calculated in Eqs 7, 8, and 9 where x_i_ denotes the observed value, Y_i_ denotes the predicted value, while the total number of items in the dataset is denoted by m. Table 4 shows that the proposed HFS-based GRU outperforms the LSTM model. Table 4 reveals the least RMSE values for Habib Bank Limited (HBL), National Bank Pakistan (NBP), United Bank Limited (UBL), and Bank Alfalah which are 14.5877, 6.437014, 6.63871, and 6.806898, respectively, using an HFS-based GRU model.


$$MSE=\left(\frac{1}{m}\right)\sum_{k=1}^{m}\left|{\left(x_{i}-\;y_{i}\right)}^{2}\right|$$ (7)

$$MAE=\left(\frac{1}{m}\right)\sum_{k=1}^{m}\left|x_{i}-\;y_{i}\right|$$ (8)

$$RMSE=\sqrt{\left(\frac{1}{m}\right)\sum_{k=1}^{m}{\left(x_{i}-\;y_{i}\right)}^{2}}$$ (9)

**Table 4:** Outcomes.

Stock Name	Prediction Model	RMSE	MAE	MSE
Habib Bank	HFS based LSTM	112.8921	117.6987	28543.56
United Bank	HFS based LSTM	18.52552	11.6681	210.6903
National Bank	HFS based LSTM	26.8476	12.07362	464.6993
NetSol Tech	HFS based LSTM	7.815948	6.612878	26.26087
Bank Alfalah	HFS based LSTM	14.77706	8.776213	162.8786
Habib Bank	HFS based GRU	14.5877	9.917682	112.7889
United Bank	HFS based GRU	6.63871	4.256876	17.91714
National Bank	HFS based GRU	6.437014	4.044718	16.71143
NetSol Tech	HFS based GRU	26.920323	23.200847	631.1598
Bank Alfalah	HFS based GRU	6.806898	6.810566	37.79903

**Fig 11:**
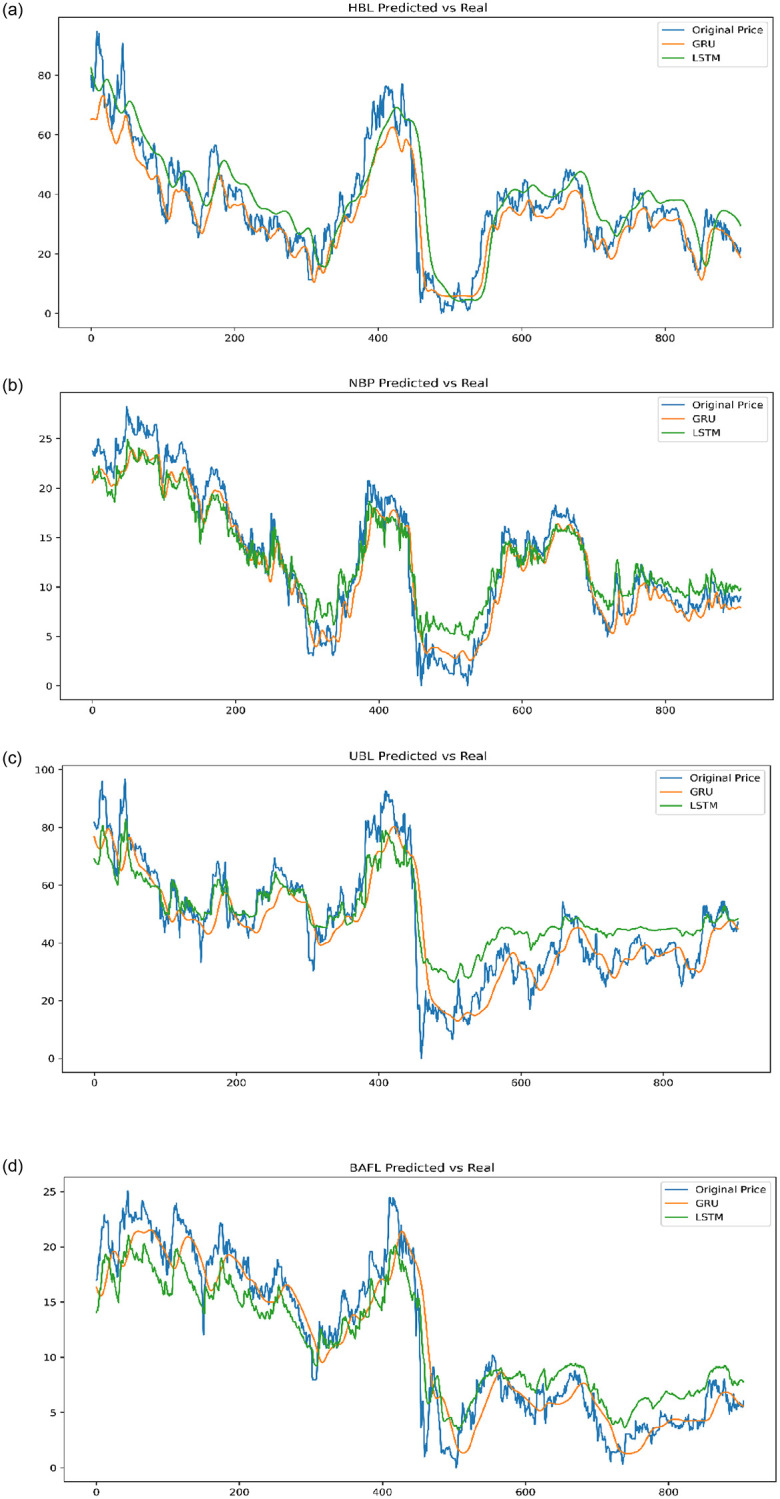
GRU and LSTM prediction. (a) HBL Bank. (b) NBP Bank. (c) UBL Bank. (d) Bank Alfalah.

As demonstrated in Fig 11, the data points for the GRU model fit better than that of the LSTM model. Friedman test [61] was used for the validation of the GRU and LSTM method’s post-processing findings, and Eq 10 defines it. The prediction model’s number is k, the total number of items is N, and R_i_ is the total of the *i* prediction model’s rankings.


$$\frac{12}{nk\left(k+1\right)}\;\sum_{i=1}^{k}R_{i}^{2}-3n\left(k+1\right)$$ (10)

To evaluate if the GRU and LSTM prediction model findings are significant, we formulated the null hypothesis and alternate hypotheses below.

*h*_a_: The GRU and LSTM prediction models get the same results.
*h*_b_: The GRU and LSTM prediction models provide different results.

The Friedman test was used to confirm the outcome for Habib Bank shares. We found that the value of chi-squared is 29.7132, the p-value is 0.0364, and df = 1. The alternative hypotheses are rejected because the p-value is less than 0.05. According to our findings, the outcomes of the GRU and LSTM prediction models for Habib Bank shares are identical. For National Bank stock, authors establish value of chi-squared as 21.312, p-value 0.0423, and df = 1. Alternative hypotheses are rejected because the p-value is less than 0.05.

We found that the outcomes of the GRU and LSTM prediction models for National Bank stock are equivalent. We found that the chi-squared value for United Bank stock is 21.2118, df = 1, and the p-value is 0.04014. The alternative hypotheses are rejected because the p-value is less than 0.05. We found that the GRU and LSTM models produced the same results for United Bank shares. We found that the chi-squared value for Allied Bank stock is 12.0074349, df = 1, and the p-value is 0.02765. The alternative hypotheses are rejected because the p-value is less than 0.05. We found that the outcomes of the GRU and LSTM prediction models for Allied Bank shares are equivalent. For Bank Alfalah stock, the authors establish the value of chi-square which is 0.0077855, the p-value is 0.0298, and the df = 1. The alternative hypotheses are rejected because the p-value is less than 0.05. We found that the outcomes of the GRU and LSTM prediction models for Bank Alfalah stock are equivalent. We found that the GRU and LSTM prediction model findings are significantly based on the Friedman statistical test.

Many factors influence stock prices, including political uncertainties, bond market rates, firm balance sheet changes, and international market movements. When there is a rapid change in management or a bonus announcement and share dividend, the prices of the stock might respond. Stock price swings in the financial market are solely dependent on several information sources. It’s difficult for understanding the data received through several sources. In the future, combining and interpreting data from several platforms will be a huge challenge.

## Theoretical and practical contribution

The present study attempts to address stock crisis prediction and in doing so makes significant contributions. First, the study extends the limited research on the understanding of factors and their impact on the stock market. The usage of the HFS method for the removal of stock’s unnecessary financial attributes. Second, The Naïve Bayes approach, on the other hand, is used for the classification of strong fundamental stocks. Third, Stochastic Relative Strength Index (StochRSI) is employed to identify a stock price bubble. Fourth, we identified the stock market crisis point in stock prices through moving average statistics. Fifth, the prediction of stock crises by using deep learning algorithms such as Gated Recurrent Unit (GRU) and Long-Short Term Memory (LSTM). Root Mean Square Error (RMSE), Mean Squared Error (MSE) and Mean Absolute Error (MAE) are implemented for assessing the performance of the models. The HFS-based GRU technique outperformed the HFS-based LSTM method to anticipate the stock crisis.

## Conclusion and future work

Identification of a stock crisis is difficult due to heightened volatility in the stock market. Based on the literature, to the best of our knowledge, this is the first method for predicting stock market crises based on financial considerations and stock prices. To eliminate extraneous stock financial parameter characteristics, we introduced the Hybridized Feature Selection method. The fundamentally strong stock is found using the NB classifier approach. The StochRSI technique is then used to identify stock over price. Stock crisis points are identified using moving average statics. The LSTM and GRU deep learning models are used to assess the proposed model’s efficacy. MSE, MAE, and RMSE are used to assess the model’s performance. The HFS-based GRU approach outperforms the HFS-based LSTM method.

As a result, new basic stock and technical factors might be used in future studies to increase the accuracy of the model. We looked at a small quantity of technical stock price characteristics. In the upcoming time, the researchers may look at additional technical signs to see if they can forecast when a crisis would occur. With a new optimizer, there is more opportunity to refine and fine-tune the GRU Model. Future studies might include parameter optimization for LSTM and GRU models utilizing evolutionary algorithms.
